# Physiological and comparative transcriptome analysis of the response and adaptation mechanism of the photosynthetic function of mulberry (*Morus alba* L.) leaves to flooding stress

**DOI:** 10.1080/15592324.2022.2094619

**Published:** 2022-07-04

**Authors:** Quan Su, Zhiyu Sun, Yuting Liu, Jiawei Lei, Wenxu Zhu, Liao Nanyan

**Affiliations:** a College of Life Science, Guangxi Normal University, Guilin, Liaoning, China; b College of Forestry, Shenyang Agricultural University, Shenyang, Guangxi, China; c Guangxi Fangcheng Golden Camellias National Nature Reserve, Guilin 541006, Guangxi, China

**Keywords:** Flooding stress, mulberry, transcriptomic, photosynthetic, chlorophyll synthesis

## Abstract

Flooding has become one of the major abiotic stresses that seriously affects plant growth and development owing to changes in the global precipitation pattern. Mulberry (*Morus alba* L.) is a desirable tree spePhysocarpus amurensis Maxim andcies with high ecological and economic benefits. To reveal the response and adaptive mechanisms of the photosynthetic functions of mulberry leaves to flooding stress, chlorophyll synthesis, photosynthetic electron transfer and the Calvin cycle were investigated by physiological studies combined with an analysis of the transcriptome. Flooding stress inhibited the synthesis of chlorophyll (Chl) and decreased its content in mulberry leaves. The sensitivity of Chl *a* to flooding stress was higher than that of Chl *b* owing to the changes of *CHLG* (LOC21385082) and *CAO* (LOC21408165) that encode genes during chlorophyll synthesis. The levels of expression of Chl *b* reductase *NYC* (LOC112094996) and *NYC* (LOC21385774), which are involved in Chl *b* degradation, were upregulated on the fifteenth day of flooding, which accelerated the transformation of Chl *b* to Chl *a*, and upregulated the expression of *PPH* (LOC21385040) and *PAO* (LOC21395013). This accelerated the degradation of chlorophyll. Flooding stress significantly inhibited the photosynthetic function of mulberry leaves. A Kyoto Encyclopedia of Genes and Genomes (KEGG) enrichment analysis of differentially expressed genes under different days of flooding stress indicated significant enrichment in Photosynthesis-antenna proteins (map00196), Photosynthesis (map00195) and Carbon fixation in photosynthetic organisms (map00710). On the fifth day of flooding, 7 and 5 genes that encode antenna proteins were identified on LHCII and LHCI, respectively. They were significantly downregulated, and the degree of downregulation increased as the trees were flooded longer. Therefore, the power of the leaves to capture solar energy and transfer this energy to the reaction center was reduced. The chlorophyll fluorescence parameters and related changes in the expression of genes in the transcriptome indicated that the PSII and PSI of mulberry leaves were damaged, and their activities decreased under flooding stress. On the fifth day of flooding, electron transfer on the PSII acceptor side of mulberry leaves was blocked, and the oxygen-evolving complex (OEC) on the donor side was damaged. On the tenth day of flooding, the thylakoid membranes of mulberry leaves were damaged. Five of the six coding genes that mapped to the OEC were significantly downregulated. Simultaneously, other coding genes located at the PSII reaction center and those located at the PSI reaction center, including Cytb6/f, PC, Fd, FNR and ATP, were also significantly downregulated. In addition, the gas exchange parameters (*P*_n_, *G*_s_, *T*_r_, and *C*_i_) of the leaves decreased after 10 days of flooding stress primarily owing to the stomatal factor. However, on the fifteenth day of flooding, the value for the intracellular concentration of CO_2_ was significantly higher than that on the tenth day of flooding. In addition, the differentially expressed genes identified in the Calvin cycle were significantly downregulated, suggesting that in addition to stomatal factors, non-stomatal factors were also important factors that mediated the decrease in the photosynthetic capacity of mulberry leaves. In conclusion, the inhibition of growth of mulberry plants caused by flooding stress was primarily related to the inhibition of chlorophyll synthesis, antenna proteins, photosynthetic electron transfer and the Calvin cycle. The results of this study provide a theoretical basis for the response and mechanism of adaptation of the photosynthetic function of mulberry to flooding stress.

## Introduction

Under the influence of global climate change, extreme precipitation, increasing groundwater levels, unreasonable irrigation measures and other factors, flooding has become one of the major sources of adversity for plants, which seriously affects social security and environmental sustainability.^1 2^ More than 1,700 M ha of land area throughout the world is seriously affected by soil flooding every year.^3^ The high incidence of flooding causes more climate-related disasters to crops, agricultural assets and infrastructure than any other abiotic factor.^4^ Flooding stress induces plants to respond at morphological and physiological levels^5 6^ and reduces growth and yield.^7 8^ The yield of wheat (*Triticum aestivum* L.) was found to decrease by approximately 43% when affected by flooding stress.^9^ The Arkansas Rice Research and Extension Center (Stuttgart, AR, USA) simulated field flooding to stress winter wheat, and the yield of winter wheat decreased by 34%.^10^ When cotton (*Gossypium hirsutum* L.) was subjected to flooding stress at different growth stages, the yield decreased in varying degrees, with a maximum yield reduction of 63%.^11^ Flooding can also lead to many changes in plants, such as the reduction of aboveground and root biomass and the absorption of root nutrients,^12 13^ the reduction of hydraulic conductivity, yellowing, necrosis and defoliation,^14 15^ and the reduction of chlorophyll content and photosynthetic performance,^16^ affect all growth stages from seed germination to vegetative and reproductive growth. Flooding can change the soil pH, redox potential, and level of hypoxia (< 21% O_2_).^17 18^ The primary challenge of flooding to plants is the energy crisis caused by the reduction of oxygen supply,^19 20^ which damages the cell and physiological functions of plants and has a negative impact on their developmental stages.^2^ Many studies have shown that mulberry can slow down surface runoff, protect soil resources, and show good adaptability to flooding.^21 22^ After 90 days of soaking, the germination rate of mulberry seedlings could reach 62.6%.^23^ Rao et al.^24^ found that during flooding, the leaves of seedlings treated with shallow submergence remained healthy, while the leaves treated with half submergence and full submergence showed different degrees of waterlogging symptoms in the middle and late flooding periods, and formed adventitious roots at the stem base.

The primary consequence of plant flooding is the reduction in plant photosynthesis. Since the diffusion of gas in water is 10,000 times slower than that in air, the limitation of O_2_ diffusion is the largest environmental factor that affects plant photosynthesis under flooding conditions.^25^ During flooding, the concentration of CO_2_ and O_2_ can determine the degree of photosynthesis and metabolism of flooded plants, which substantially affects their physiological states.^26^ Under hypoxic conditions, carbohydrate consumption will lead to the risk of carbohydrate reserve depletion and oxidative damage, which will result in the consumption of large quantities of energy by the plant.^27^ In many plants, hypoxia first leads to the closure of leaf stomata, increases the resistance of CO_2_ diffusion to leaves,^28^ and then affects the activities of photosynthetic-related enzymes,^29^ reducing the content of chlorophyll^15^ and its photosynthetic capacity.^30–32^ The decrease of carboxylation efficiency and photochemical activity could also be the reason for the decrease in rate of plant photosynthesis under flooding.^33^ He et al.^34^ and Barickman et al.^35^ found that hypoxia limited the gas exchange parameters of plants and led to the impairment of photosynthetic activity. In a study by Zafa,^2^ hypoxia stress reduced the photosynthetic pigments, stomatal conductance, intercellular CO_2_ concentration and photosynthetic activity of plant leaves. The duration of flooding stress affected the damage of PSII structure and activity, as well as the development of plant photosynthetic regulation.^36^ Caroline et al. ^37^ found that the photosynthetic performance index showed that the stress led to a gradual decrease in parameters on the third day of flooding, indicating that photosystem II was damaged. When *Capsicum annuum* var. *glabriusculum* was subjected to flooding stress, Martinez-Acosta et al. ^38^ found that flooding had a negative effect on stomatal conductance, the photosynthetic rate and transpiration rate from 20 d after transplanting, and the biomass was significantly reduced at 120 d. The negative effects of flooding on vegetative growth are also associated with low photosynthetic rates in the Solanaceae.^39^ However, the fluorescence parameters of mulberry showed a relatively stable trend in the late flooding period.^24^

*Morus alba* L. is a perennial deciduous tree in the Moraceae that is economically important in China. It is considered to be an excellent tree with important ecological and economic benefits. Simultaneously, mulberry is strongly adaptable to abiotic stresses, such as salinity and drought, and also has strong anti-seasonal flooding resistance. Currently, research on mulberry flooding stress primarily focuses on the growth and survival rate of mature mulberry trees under water stress,^23 40 41^ but there are no reports on the response of photosynthetic function of mulberry leaves to flooding stress. Transcriptomics is one of the most important methods to analyze the level of changes in genes in plants under stress, and it plays an important role in revealing the molecular mechanism of plant adaptation under stress.^42 43^ Therefore, mulberry seedlings were selected as experimental materials in this study. Plant physiology combined with transcriptome methods were used to study the response and adaptation mechanism of chlorophyll synthesis and degradation, PSII and PSI photochemical activities, PSII donor side and receptor side electron transport, photosynthetic antenna protein, photosynthetic gas exchange parameters and Calvin cycle to flooding stress. This study provides new insights to understand the molecular mechanism of mulberry responses and adaptation to flood stress.

## 1. Materials and methods

### 1.1 Plant materials and treatment

The annual seedling *Morus alba* was selected as the experimental material. After germination and cultivation, seedlings that had grown to 20 cm were transplanted into a culture bowl with a diameter of 33 cm that were 28 cm high. The culture substrate was evenly mixed with peat soil and vermiculite. To ensure the relative consistency of the test materials, the branches and leaves of mulberry seedlings were removed during transplantation, and only the main root and main stem were kept at 5 cm each. Two plants were planted in each pot. When the seedlings tested grew to approximately 40 cm, 80 pots of seedlings with relatively consistent rates of growth were selected for the experimental treatment. Four treatment groups were designed, and 20 culture bowls were repeated for each treatment. Mulberry trees that were not subjected to waterlogging treatment were established as the waterlogging control (CK) and were watered daily with tap water to maintain approximately 70% of the soil moisture holding capacity. In the flooded treatment group, the water level was always 5 cm above the soil surface. In order to ensure the consistency of seedling growth days, the reverse order method was adopted for flooding treatment. After 15 days of flooding treatment, mulberry leaves of the CK group, 5-day flooding treatment group (D5), 10-day flooding treatment group (D10) and 15-day flooding treatment group (D15) were randomly sampled to determine the following physiological and transcriptome indices.

### 1.2 Determination of parameters

**Determination of chlorophyll content**: Fresh leaves without main veins were soaked in a solution of acetone and ethanol (1:1 [v/v]) to extract the pigments. The contents of chlorophyll a (Chl *a*), chlorophyll b (Chl *b*), chlorophyll a/b (Chl *a*/*b*) and chlorophyll a + b (Chl *a* + *b*) were calculated as described by Porra.^44^

**Determination of gas exchange parameters**: The net photosynthetic rate (*P*_n_), stomatal conductance (*G*_s_), transpiration rate (*T*_r_) and intercellular CO_2_ concentration (*C*_i_) of the third fully expanded leaves from the top to bottom were measured using a portable photosynthetic measurement system (LICOR, Lincoln, NE, USA). During the measurement process, a LICOR-6400 self-equipped light source and CO_2_ cylinder were used to establish the light intensity to 800 μmol m^−2^·s^−1^. The concentration of CO_2_ in the fixed system was 400 cm^3^·m^3^.

**Determination of the OJIP curve and 820 nm light reflection curve (*MR*_820_**): The fully expanded functional leaves on mulberry plants with different treatments were selected, and the dark adaptation clip was used for 30 min of dark adaptation. The OJIP curve and 820 nm light reflection curve (*MR*_820_) of the leaves after dark adaptation were measured using a Hansatech Multifunctional Plant Efficiency Analyzer (M-PEA; Hansatech Instruments, Ltd., King’s Lynn, UK), and the measurements were repeated five times. The corresponding time points of O, J, I and P on the OJIP curve were 0.01, 2, 30 and 1000 ms, respectively, represented by *F*_o_, *F*_J_, *F*_I_ and *F*_p_, respectively. Point L represents the corresponding point on the curve at 0.15 ms, and point K represents the corresponding point on the curve at 0.3 ms. The O-P, O-J and O-K on the OJIP curve were standardized, i.e., the relative fluorescence intensity (*F*_o_) of the O point was set to 0, and the *F*_p_ of the P, J and K points were set to 1. The standardized formula is as follows: *V*_O-P_ = (*Ft-F*_o_)/(*F*_p_-*F*_o_), *V*_O-J_ = (*F*_t_-*F*_o_)/(*F*_J_-*F*_o_) and *V*_O-K_ = (*F*_t_-*F*_o_)/(*F*_K_-*F*_o_) where *F*_t_ represents the relative fluorescence intensity at each time point. The relative variable fluorescence intensity of the L, K and J points on the normalized curve was expressed as *V*_L_, *V*_K_ and *V*_J_ where *V*_L_ = (*F*_L_-*F*_o_)/(*F*_K_-*F*_o_), *V*_K_ = (*F*_K_-*F*_o_)/(*F*_J_-*F*_o_) and *V*_J_ = (*F*_J_-*F*_o_)/(*F*_P_-*F*_o_), respectively. A JIP-test analysis was conducted as calculated by Strasser.^45^ The maximum photochemical efficiency of PSII is *F*_v_/*F*_m_; the photosynthetic performance index based on light absorption is *PI*_ABS_, and the performance index based on unit area is *PI*_total_. The activity of PSI reaction center was reflected by the slope of the initial section of the *MR*_820_ curve, namely Δ*I*/*I*_o_, where *I*_o_ and Δ*I* represent the maximum and the difference between the maximum and minimum reflected signals in the 820 nm light reflection curve, respectively.^46 47^

**Determination of transcriptome**: The leaves of mulberry trees were cut and wrapped in tinfoil and then frozen with liquid nitrogen for 30 min. The plant samples were sent to Shanghai Majorbio (Shanghai, China) in an incubator with dry ice for transcriptome data analysis. The main process was as follows:

(1). Sequencing experiment process

①. Extraction of total RNA: Total RNA was extracted from tissue samples. A NanoDrop2000 (Thermo Fisher, Waltham, MA, USA) was used to detect the concentration and purity of the extracted RNA. Agarose gel electrophoresis was used to detect the integrity of RNA, and an Agilent 2100 (Agilent Technologies, Santa Clara, USA, CA) was used to determine the RNA Integrity (RIN) value. RNA quantity >1 ug, concentration >35 ng·μL^−1^, A260/280 > 1.8, and A260/230 > 1.0 were required for single library construction. ②. Oligo dT enriched mRNA: Eukaryotic mRNA has a polyA tail structure at the 3’ end, and a T base pairing with polyA by magnetic beads with Oligo (dT) can be used to isolate the mRNA from total RNA to analyze transcriptome information. ③. Segmented mRNA: An Illumina NovaSeq 6000 platform (Illumina, San Diego, CA, USA) is designed to sequence short sequences. The enriched mRNA is a complete RNA sequence with an average length of several KB, which requires random interruption. The mRNA can be randomly fractured by fragmentation buffer, and small fragments of approximately 300 bp can be separated by magnetic bead screening. ④. Reverse synthesis of cDNA: Under the action of reverse transcriptase, random hexamers were added to reverse synthesize the first-strand cDNA using mRNA as a template, and the second strand was synthesized to form a stable double-strand structure. ⑤. Adaptor: The double-stranded cDNA structure is the viscous end. An End Repair Mix was added to fill the flat end, and an “A” base was then added to the 3’ end to connect the Y-shaped connector;⑥. Sequencing on the Illumina platform: The library was enriched, and 15 cycles were amplified by PCR. Electrophoresis on a 2% agarose gel was used to recover the target band. TBS380 (PicoGreen) quantitative, according to the data proportion mixed machine. Bridge PCR amplification was performed on cBot to generate clusters. Illumina platform sequencing (PE library, read length 2 × 150 bp).

(2). Quality control data statistics

The quality of subsequent assembly will be seriously affected because the original sequencing data contained sequencing joint sequences, low-quality reads, sequences with high uncertain base information rate and excessively short sequences. To ensure the accuracy of subsequent bioinformation analysis, the original sequencing data were filtered to obtain high-quality clean data to ensure the smooth progress of subsequent analyses. The specific steps and sequences were as follows:① The connector sequence in reads was removed, and the reads without inserted fragments owing to connector self-connection and other reasons were also removed. ②. The bases were trimmed at the 3’ end of the sequence with low quality (mass value < 30). If there were still bases with a mass value < 10 in the remaining sequence, the whole sequence would be eliminated. ③. Reads with uncertain base information ratio exceeding 10% were removed. ④. Sequences that were < 50 bp long were removed after removing the adapter and quality pruning. The software used included SeqPrep ((https://github.com/jstjohn/SeqPrep) and Sickle (https://github.com/najoshi/sickle)

(3). Differential gene screening

Expression difference analysis: Based on the quantitative results of expression, the differentially expressed genes (DEGs) between the two groups were analyzed, and the DEGs between the two groups were obtained. The differential analysis software was deseq2, and the screening threshold was log_2_FC>1 and *P*< 0.05.

(4). Function annotation statistics

①. GO enrichment analysis: The software Goatools (https://github.com/tanghaibao/GOatools) was used to conduct a Gene Ontology (GO) enrichment analysis of the concentration of genes. The default to correct the P-values when *P* < 0.05, enrichment of thought plays a significant role in the function of the GO.

②. KEGG pathway enrichment analysis: A Kyoto Encyclopedia of Genes and Genomes (KEGG) pathway enrichment analysis was performed on genes/transcripts in gene concentrations using scripts written in R language. The principle of calculation was the same as that used in the GO functional enrichment analysis. By default, when P < 0.05, the KEGG pathway was considered to be significantly enriched.

### 1.3 Statistical Analysis

Microsoft Excel 2016 (Redlands, WA, USA) and SPSS 22.0 (IBM, Inc., Armonk, NY, USA) were used to analyze the data, and a one-way analysis of variance (ANOVA) and least significant difference method (LSD) were used to compare differences between different data groups.

## 2. Results

### 2.1 Differentially expressed genes (DEGs)

On the fifth and tenth day of flooding, 2,162 DEGs (up 1,109, down 1,053) and 2,197 DEGs (up 1,218, down 1,079) were identified compared with the CK, respectively, indicating that there was no significant difference in the number of DEGs between the fifth and tenth day of flooding (Figure 1 -A, B, D). However, on the fifteenth day of flooding, the number of DEGs increased significantly, and 3,755 DEGs were identified, including 1,947 upregulated DEGs and 1,808 downregulated DEGs, indicating that more of the downregulated DEGs increased (Figure 1 -C, D). A total of 894 DEGs had common changes under different days of flooding. This was approximately half of the number of DEGs on the fifth and tenth days of flooding. However, 2,037 DEGs with specific changes were reached on the fifteenth day of flooding; this was not only a significant increase but also revealed specific changes (Figure 1 -E). A PCA analysis between samples showed that the three biological replicates of the same treatment clustered together, indicating that the samples were highly repeatable. However, the two treatments were distributed in different quadrants with the CK and 15 d of flooding except that the treatments at 5 d and 10 d of flooding were distributed in the same quadrant (Figure 1 -F). A cluster analysis of the heatmap in Figure 1 -G indicated that the three biological replicates of the 12 samples (four treatments and three biological replicates) all clustered together. Three CK groups and three flooded fifth day treatment groups were in clade 1, while three flooded tenth day treatment groups and flooded fifteenth day treatment groups were in clade 2. This indicated that the difference between the changes of mulberry DEGs and the CK was relatively small on the fifth day of flooding, while on the tenth and fifteenth days of flooding, although the number of DEGs varied greatly, the trends of changes in them were similar.

**Figure 1.**
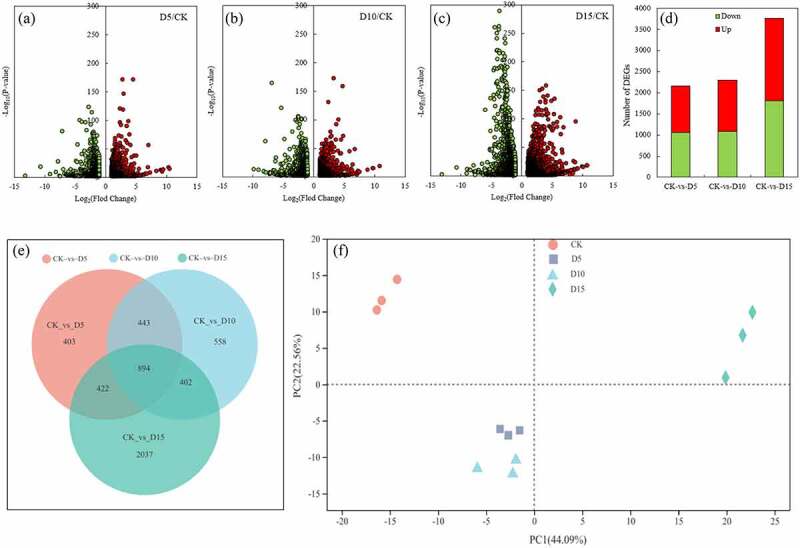
Volcano plot of DEGs (A, B, C), number of DEGs (D), Venn diagrams of DEGs (E), PCA (F) and heatmap of DEGs (G) in mulberry (*Morus alba* L.) leaves under flooding stress. DEGs, differentially expressed genes; PCA, principal component analysis.

**Figure 1.**
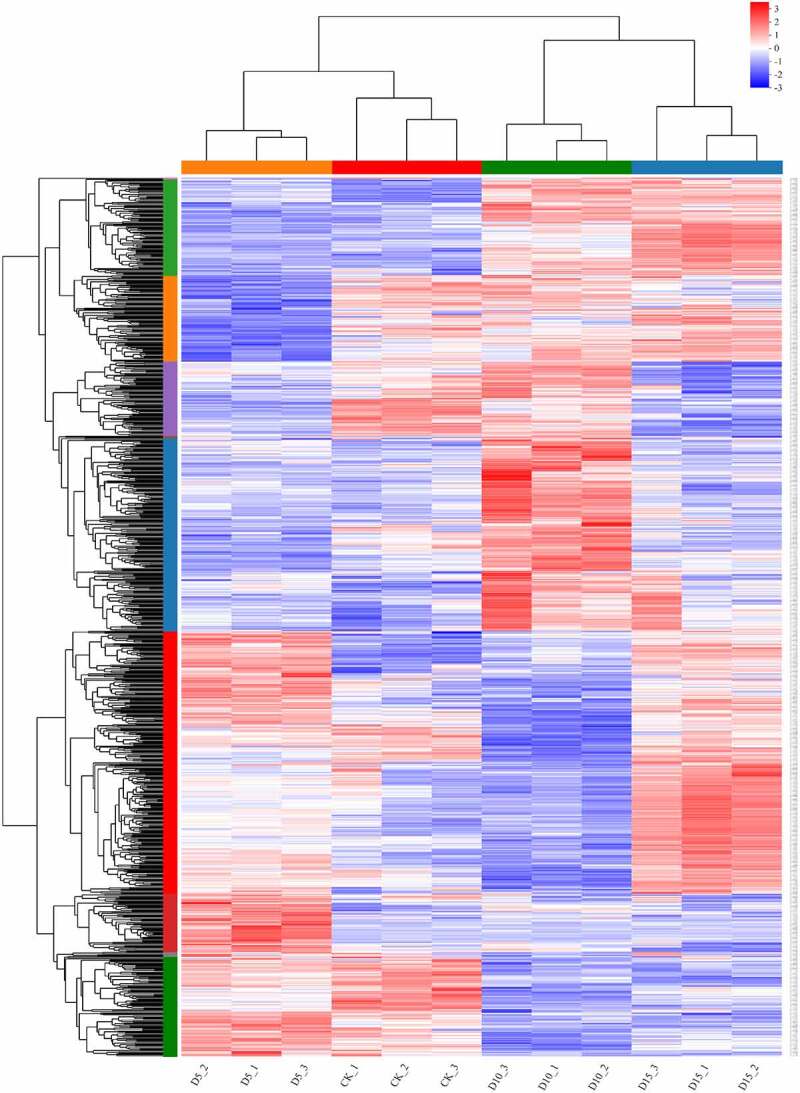
(continued)

### 2.2 KEGG analysis of the DEGs

**Figure 2.**
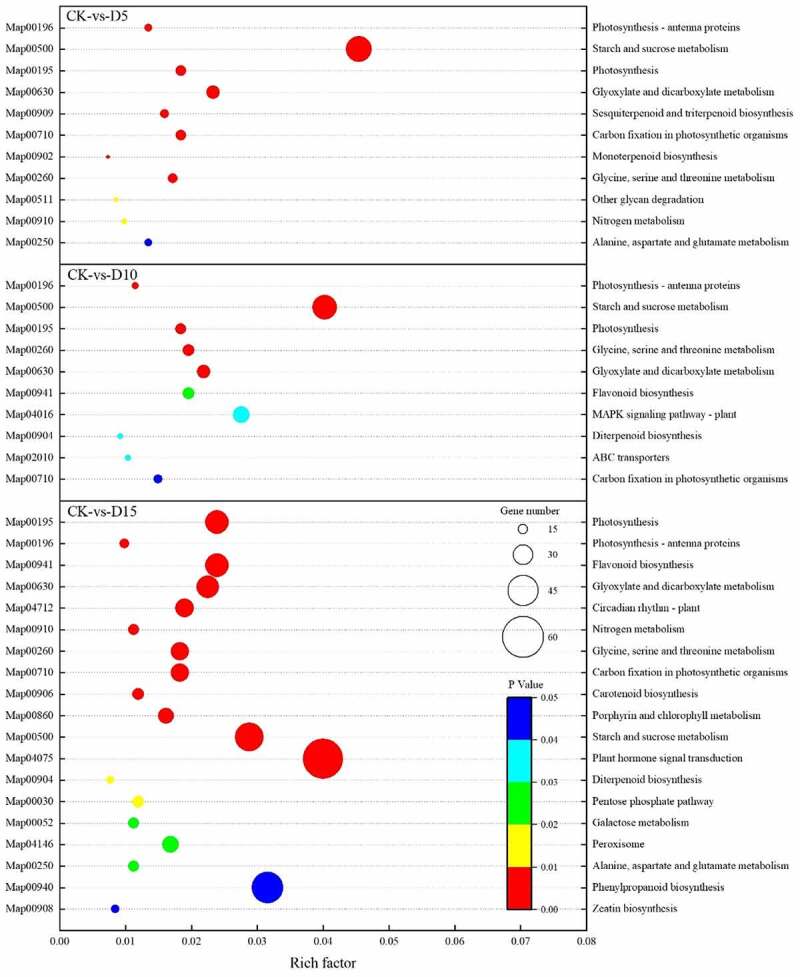
Kyoto Encyclopedia of Genes and Genomes (KEGG) pathway enrichment analysis of DEGs in mulberry (*Morus alba* L.) leaves under flooding stress. DEGs, differentially expressed genes.

An analysis of the KEGG enrichment pathways with P < 0.05 showed that 11 and 10 KEGG pathways were significantly enriched on the fifth and tenth day of flooding, respectively, while the number of KEGG pathways that were significantly enriched increased to 19 on the fifteenth day of flooding (Figure 2). Five KEGG pathways were significantly enriched in different days of flooding, including Photosynthesis-antenna proteins (map00196), Photosynthesis (map00195), Starch and sucrose metabolism (map00941) and Carbon fixation in photosynthetic organisms (map00710), Glycine, serine, and threonine metabolism (map00260) and significantly enriched in the Nitrogen metabolism pathway (MAP00910) on the fifth and fifteenth days of flooding. In brief, the flooding stress significantly affected the photosynthesis, carbon assimilation and metabolism, and amino acid metabolism in the nitrogen metabolism of mulberry leaves. In addition, under different flooding days, the photosynthesis antenna proteins (map00196) in the KEGG pathway were ranked first, first and second, respectively, based on the *P*-values from small to large, and photosynthesis (map00195) was ranked third, third and first, respectively, based on the *P*-values from small to large. These results indicate that the capture of light energy and photosynthetic electron transfer by antenna proteins were the processes that were the most sensitive to flooding. Next, we focused on a quantitative analysis of changes in the levels of expression of the enzymes that encode genes in photosynthesis-related processes, such as chlorophyll synthesis, antenna proteins, photosynthetic electron transport and the Calvin cycle.

### 2.3 Chlorophyll content and expression of the genes for chlorophyll synthesis and degradation

**Figure 3.**
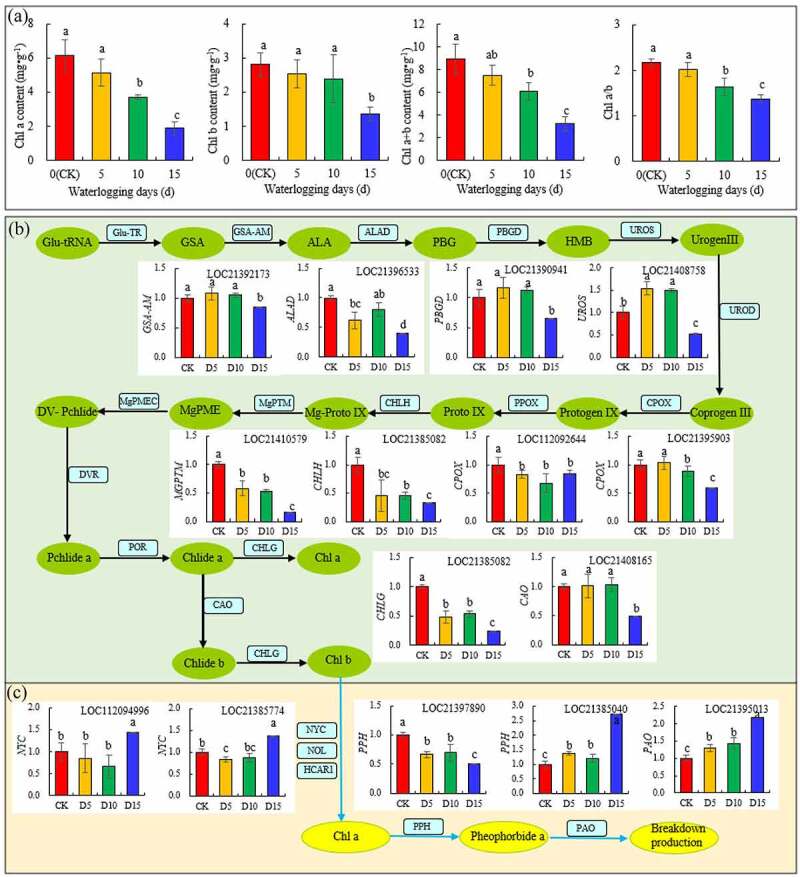
Chlorophyll a (Chl a), chlorophyll b (Chl b), chlorophyll a + b (Chl a + b) content and chlorophyll a/b (Chl a/b) ratio (A) and expression of the genes of chlorophyll synthesis (B) and chlorophyll degradation (C) in mulberry (*Morus alba* L.) leaves under flooding stress.

In Figure 3 -A, the chlorophyll content of mulberry leaves decreased with the increase in days of flooding, but on the fifth day of flooding, the Chl *a*, Chl *b* and Chl *a*+ *b* contents of mulberry leaves decreased slightly compared with the CK, but the differences were not significant. On the tenth day of flooding, the contents of Chl *a* and Chl *a*+ *b* decreased significantly compared with the CK, but the contents of Chl *a*, Chl *b* and Chl a + b decreased significantly on the fifteenth day of flooding. Similar to Chl *a* and Chl *a*+ *b*, Chl *a*/*b* in the mulberry leaves decreased by 25.06% (*P*< 0.05) compared with the CK on the tenth day of flooding and decreased by 37.02% (*P*< 0.05) compared with the CK on the fifteenth day of flooding. Thus, the sensitivity of mulberry leaves Chl *b* to flooding stress was lower than that of Chl *a*.

Ten coding genes that changed significantly during the synthesis of chlorophyll by mulberry leaves under flooding stress were identified (Figure 3 -B). The levels of expression of *GSA-AM* (LOC21392173), *PBGD* (LOC21390941) and *CHLG* (LOC21385082) were only significantly downregulated on the fifteenth day of flooding stress compared with the CK. The levels of expression of *CPOX* (LOC112092644), *CHLH* (LOC21385082), *MGPTM* (LOC21410579) and *CAO* (LOC21395903) decreased significantly from the fifth day of flooding. The level of expression of *CPOX* (LOC21395903) decreased significantly on the tenth day of flooding. The level of expression of the *UROS* (LOC21408758) gene increased on the fifth and tenth days of flooding and decreased on the fifteenth day of flooding.

Five coding genes changed significantly during the degradation of chlorophyll. During the transformation from Chl *b* to Chl *a*, the levels of expression of the *NYC* (LOC112094996) and *NYC* (LOC21385774) genes increased significantly on the fifteenth day of flooding. Although the levels of expression of *PPH* (LOC21397890) were downregulated after flooding, the levels of expression of *PPH* (LOC21385040) and *PAO* (LOC21395013) were significantly upregulated on the fifth day of flooding and increased more than twice as much as those of the CK on the fifteenth day of flooding (Figure 3 -C).

### 2.4 PSII and PSI photochemical activity

**Figure 4.**
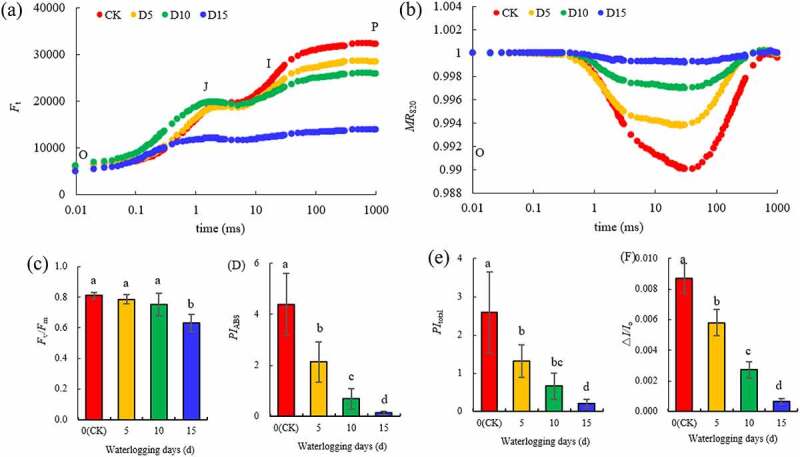
OJIP curve (A), *MR*_820_ curve (B), *F*_v_/*F*_m_ (C), *PI*_ABS_ (D), *PI*_total_ (E), Δ*I/I*_o_ (F) in mulberry (*Morus alba* L.) leaves under flooding stress.

In Figure 4 -a, the relative fluorescence intensity (*F*_t_) of mulberry leaves on the OJIP curve from the O to I point did not change significantly compared with the CK on the fifth day of flooding, but the *F*_t_ from the I to P point significantly decreased compared with the CK. On the tenth day of flooding, *F*_t_ increased from the O to J point in different degrees compared with the CK and increased the most particularly at the K point (0.3 ms), and the relative fluorescence intensity of the P point decreased significantly compared with the CK on the fifth day of flooding. On the fifteenth day of flooding, although the relative fluorescence intensity of O point did not change significantly compared with other treatments, Ft from the K point was significantly lower than that of other treatments, and the OJIP curve became calmer. With the increase in days of flooding, the amplitude of the *MR*_820_ curve of mulberry leaves decreased gradually compared with the CK; particularly on the fifteenth day of flooding, the *MR*_820_ curve basically changed to a straight line (Figure 3 -B). Compared with the CK, the *F*_v_/*F*_m_ of mulberry leaves did not differ significantly on the fifth and tenth days of flooding and only decreased by 22.13% (*P* < 0.05) on the fifteenth day of flooding (Figure 3 -C). However, *PI*_ABS_, *PI*_total_ and Δ*I*/*I*_o_ all decreased significantly from the fifth day of flooding and with the extension in flooding time. Compared with the CK, *PI*_ABS_, *PI*_total_ and Δ*I*/*I*_o_ all decreased by 96.99% (*P*< 0.05), 91.72% (*P*< 0.05) and 92.36% (*P*< 0.05), respectively, on the fifteenth day of flooding (Figure 3 -D – F).

### 2.5 PSII donor side and acceptor side photosynthetic electron transfer

**Figure 5.**
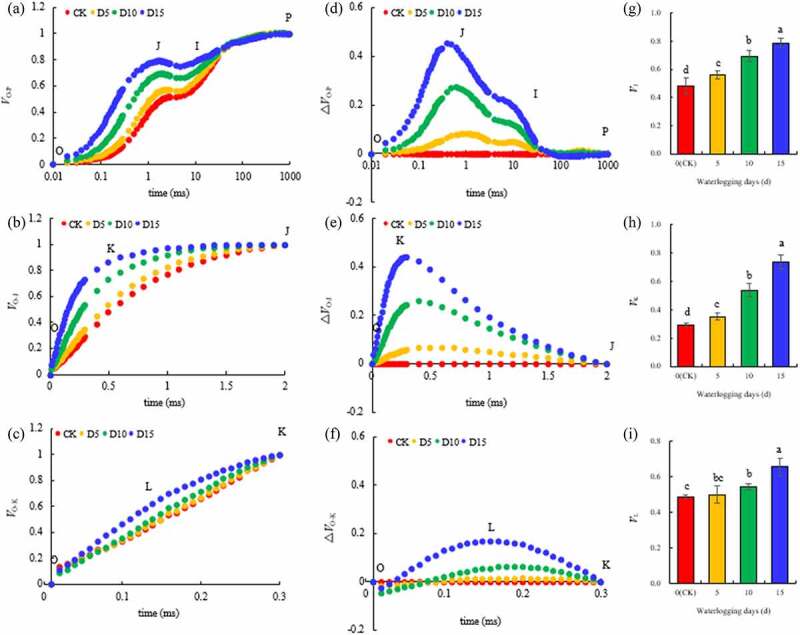
Standardized O-P（*V*_O-P_）, O-J(*V*_O-J_) and O-K curve(*V*_O-K_) (A, B, C), difference between the standardized O-P(∆*V*_O-P_), O-J(∆*V*_O-J_), and O-K curve(∆*V*_O-K_)and the CK (D, E, F), and the value of *V*_J_ (G), *V*_K_ (H) and *V*_L_ (I) in mulberry (*Morus alba* L.) leaves under flooding stress.

The OJIP curve was normalized by O-P, and the relative fluorescence intensity *V*_J_ of point J was found to change the most obviously. The O-J and O-K curves were then standardized, respectively (Figure 5). The difference between the relative fluorescence intensity *V*_K_ of point K at 0.3 ms on the O-J curve and the relative fluorescence intensity *V*_L_ of point L at 0.15 ms on the O-K curve were found to be the most obvious. Therefore, *V*_J_, *V*_K_ and *V*_L_ were quantitatively analyzed. Compared with the CK, the *V*_J_, *V*_K_ and *V*_L_ of mulberry leaves flooded for 5 days increased by 16.39% (*P*< 0.05), 19.86% (*P*< 0.05) and 2.46% (*P*> 0.05), respectively. With the extension in flooding time, the increase of the three parameters substantially increased. They increased significantly by 43.98%, 84.25% and 10.86%, respectively, by the tenth day of flooding. On the fifteenth day of flooding, they increased significantly by 63.49%, 151.37% and 34.43%, respectively.

**2.6 Levels of expression of the genes that encode photosynthetic antenna proteins and photosynthetic electron transport-related proteins**

**Figure 6.**
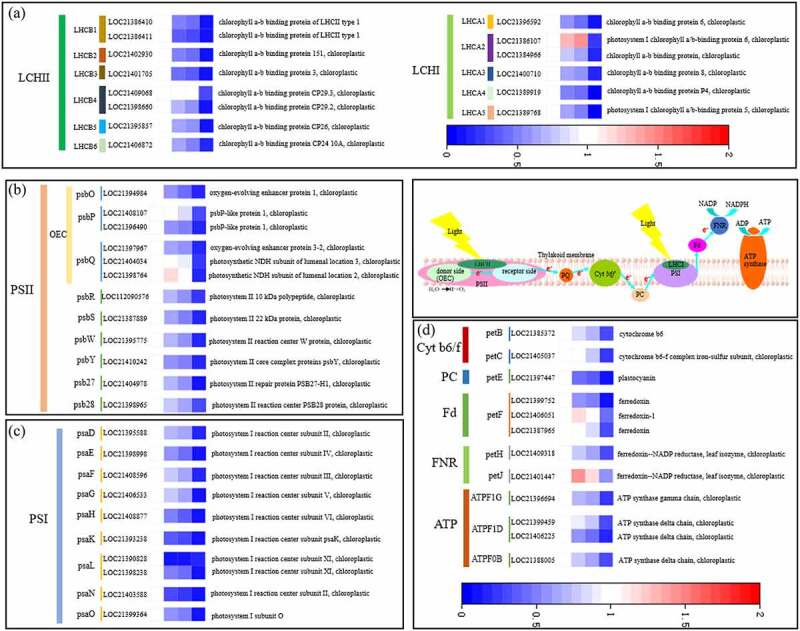
Levels of expression of the genes for the photosynthetic antenna proteins (A), PSI I (B), PSI (C) and photosynthetic electron transport (D) proteins in mulberry (*Morus alba* L.) leaves under flooding stress. PSI I, photosystem I.

In Figure 6 -A, the levels of expression of 14 coding genes of the Photosynthesis-antenna proteins were identified as significantly changed under flooding stress. Eight and six of these genes were located at LHCII and LHCI, respectively. On the fifth and tenth days of flooding, except that the expression of *LHCB4* (LOC21409068) was not significantly different from the CK, and the expression of *LHCA2*(LOC21386107) was upregulated compared with the CK, the levels of expression of two *LHCB1* and one *LHCB2, LHCB3, LHCB4, LHCB5* and *LHCB6* were significantly downregulated. The levels of expression of *LHCA1, LHCA2, LHCA3, LHCA4* and *LHCA5* in LHCI were significantly downregulated. On the fifteenth day of flooding, the 14 LHCII genes described above were significantly downregulated compared with the CK, and the range of downregulation was significantly greater than that on the fifth and tenth days of flooding.

As shown in Figure 6 -B and C, there were significant changes in the coding genes of the 12 and 10 core proteins in the PSII and PSI reaction centers of mulberry leaves under flooding stress, respectively, primarily including *PsbO, PsbP, PsbQ, PsbR, PsbS, PsbW, PsbY, Psb27* and *Psb28* located in the PSII reaction center, and *PsaD, PsaE, PsaF, PsaG, PsaH, PsaK, PsaL, PsaN* and *PsaO* located in the PSI reaction center. With one exception, the level of expression of *PsbQ* (LOC21398764) was upregulated compared with the CK on the fifth day of flooding, and the levels of expression of the other genes were downregulated. The exception was that the levels of expression of *PsbP* (LOC21408107) and *PsbQ* (LOC21404034) did not differ significantly from the CK. On the tenth day of flooding, only the level of expression of *PsbQ* (LOC21398764) did not differ significantly from the CK. On the fifteenth day of flooding, the coding genes of the 22 core proteins described above were significantly downregulated. In addition, two core protein coding genes were identified on Cyt b6/f, one on PC, three on FD, two on FNR and four in ATP synthase under flooding stress (Figure 6 -D). On the fifth day of flooding, the levels of expression of *PetC* (LOC21405037) and *PetF*(LOC21387955) did not differ significantly from the CK, and the levels of expression of *PetF* (LOC21406051) and *PetJ* (LOC21401447) were significantly upregulated. The coding genes of the other eight core proteins were significantly downregulated. On the tenth day of flooding, the level of expression of *PetF* (LOC21406051) was not changed significantly compared with the CK, and the level of expression of *PetJ* (LOC21401447) was upregulated compared with the CK. However, the coding genes of other 10 core proteins were downregulated. On the fifteenth day of flooding, all 12 coding genes were significantly downregulated.

**2.7 Photosynthetic gas exchange parameters and the levels of expression of key genes that encode enzymes in the Calvin cycle**

**Figure 7.**
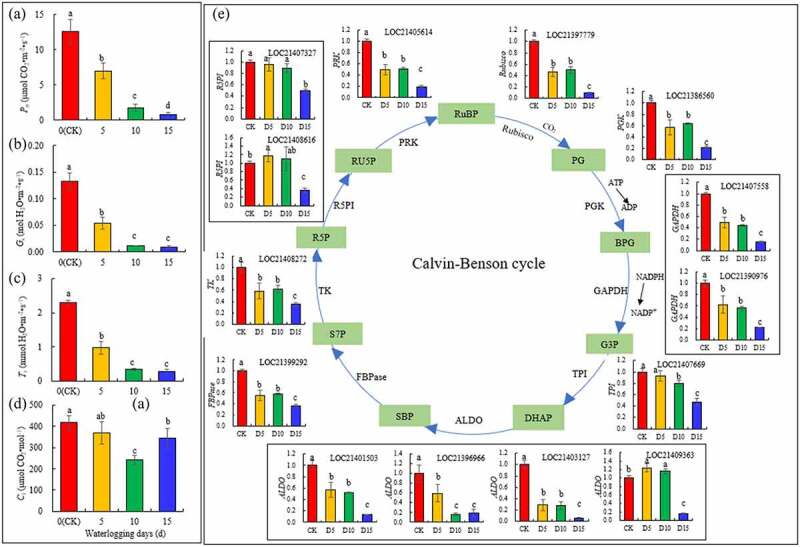
Photosynthetic gas exchange parameters (A) and levels of expression for the genes that encode key enzymes in the Calvin cycle (B) in mulberry (*Morus alba* L.) leaves under flooding stress.

In Figure 7 -A-C, *P*_n_, *G*_s_ and *T*_r_ of the mulberry leaves decreased as the number of flooding days increased, and at the fifteenth day of flooding, the three parameters decreased by 94.23% (*P*< 0.05), 93.18% (*P*< 0.05) and 89.49% (*P*< 0.05), respectively, compared with the CK. The trend of variation of *C*_i_ differed from the parameters described above. On the fifth day of flooding, there was no significant change compared with the CK. On the tenth day of flooding, it decreased by 85.30% (P < 0.05). On the fifteenth day of flooding, it decreased by 17.83% (P < 0.05) compared with the CK, but it was significantly higher than that on the tenth day of flooding (Figure 7 -D).

In Figure 6 -E, the genes that encode 14 key enzymes in the Calvin cycle were identified to change significantly under flooding stress. On the fifth day of flooding, except that the levels of expression of the *R5PI* (LOC21407327) and *TPI* (LOC21407669) genes did not differ significantly from the CK, and the levels of expression of *R5PI* (LOC21403616) and *ALDO* (LOC21409363) were significantly upregulated compared with the CK, the levels of expression of the other 10 genes were significantly downregulated. On the tenth day of flooding, except for *R5PI* (LOC21407327) and *R5PI* (LOC21408616) compared with the CK, and *ALDO* (LOC21409363) was significantly upregulated compared with the CK, the levels of expression of the other 11 genes encoded were significantly downregulated. On the fifteenth day of flooding, the levels of expression of the 14 key genes that encode the enzymes in Calvin cycle were significantly downregulated compared with the CK.

## 3.Discussion

Chlorophyll is the material basis of photosynthesis in plant leaves, and a reduction in the chlorophyll content will inhibit the capture and utilization of light energy by plants.^48^ Flooding stress inhibits the synthesis of chlorophyll in plant leaves.^15 38 49^ Related research found that the contents of Chl *a*+ *b* and Chl *b* in cucumber (*Cucumis sativus* L.) leaves decreased significantly after 10 days of flooding.^35^ Anee et al.^50^ also found that the contents of Chl *a*, Chl *b* and Chl *a*+ *b* in sesame (*Sesamum indicum* L. cv. BARI Til-4) leaves were significantly lower than those in the control after 2, 4, 6 and 8 days of flooding stress treatment. In this study, on the tenth day of flooding, the chlorophyll content of the leaves decreased. The contents of Chl *a* and Chl *a*+ *b* decreased significantly compared with the CK, but the content of Chl *b* did not change significantly. On the fifteenth day of flooding, the contents of Chl *a*, Chl *b* and Chl *a*+ *b* decreased significantly. Similar to Chl *a* and Chl *a*+ *b*, Chl *a*/*b* of mulberry leaves decreased by 25.06% and 37.02% on the tenth and fifteenth days of flooding, respectively, compared with the CK, indicating that Chl *b* of mulberry leaves is less sensitive to flooding stress than Chl *a*. To analyze the causes of this phenomenon, we quantitatively analyzed the levels of expression of the enzymes involved in chlorophyll synthesis and chlorophyll degradation in mulberry leaves. In general, more than 17 enzyme genes are involved in the Chl biosynthetic pathway in higher plants.^51 52^ Flooded conditions inhibited the expression of the genes related to chlorophyll synthesis.^53^ In our study, 10 DEGs involved in Chl biosynthesis in mulberry leaves changed significantly under flooding stress. The level of expression of *CHLG* (LOC21385082) was significantly downregulated on the fifth day of flooding, while the level of expression of *CAO* (LOC21408165) did not change significantly on the fifth and tenth day of flooding and began to be downregulated on the fifteenth day of flooding. Both Chl *a* and Chl *b* use chlorophyllide *a* as precursors. Chlorophyllide *a* forms Chl *a* directly under the action of chlorophyll synthase (CHLG), while Chl *b* forms chlorophyllide *b* under the action of chlorophyllide a oxygenase (CAO), and then Chlorophyllide *b* forms Chl *b* under the action of CHLG.^54^ Therefore, it shows that the sensitivity of mulberry leaf Chl *b* to flooding stress was lower than Chl *a* because the level of expression of the *CAO* gene changed less. Edwards et al. ^55^ also found that *Eleocharis cellulose* Torr. (Cyperaceae) decreased the contents of total chlorophyll and Chl *a* in leaves under flooding stress, while the changes of Chl *b* were not significant. Alternatively, chlorophyll b reductase *NYC* (LOC112094996) and *NYC* (LOC21385774) involved in chlorophyll b degradation began to be upregulated on the fifteenth day of flooding, accelerating the transformation of Chl *b* to Chl *a*, which explains why the content of Chl *b* began to decrease in mulberry leaves on the fifteenth day of flooding. Salah et al., ^56^ and Mahdavian et al., ^57^ also found that flooding stress resulted in a decrease in the levels of chlorophyll and an imbalance in the transformation of Chl *a* to Chl *b*. PPH and PAO are key enzymes involved in the metabolism of chlorophyll degradation.^58 59^ The role of PPH enzyme is to convert magnesium deoxychlorophyll a to magnesium deoxychlorophyllic acid.^60^ PAO is encoded by a gene that accelerates cell death,^61^ and pheide a is eventually catalyzed by PAO to form a monopyrrole oxidative degradation product.^62^ The levels of expression of *PPH* (LOC21385040) and *PAO* (LOC21395013), the key enzymes that lead to chlorophyll degradation and metabolism, were upregulated on the fifteenth day of flooding, which accelerated chlorophyll degradation and directly affected the capture of light energy.^63^ Large arrays of light-harvesting complex (LHC) are assembled as the peripheral components of PSII and PSI, designated LHCII and LHCI, respectively.^64 65^ The most important function of the LHC is to capture the solar energy and transfer its energy to the reaction center.^66 67^ The transcriptome results of this study indicated that the most significantly enriched KEGG pathway of mulberry under flooding stress was photosynthesis-antenna protein (map00196). After 5 days of flooding stress, seven of the eight antenna protein genes located on LHCII were significantly downregulated, and five of the six antenna protein genes located on LHCI were significantly downregulated. In addition, the degree of downregulation increased with the extension of flooding stress time. A study by Lee et al., ^68^ also found that when the seedlings of rape (*Brassica napus* L.) were flooded for 3 days, the levels of expression of LHCII type I and type II chlorophyll a/b-binding proteins were downregulated.

Plant photosynthesis is one of the processes that is the most sensitive to stress.^69 70^ Similar to most abiotic stresses, flooding stress also reduces the photosynthetic capacity of plants, primarily in the reduction of photochemical activity and ability to assimilate carbon ability.^2 33^ Chlorophyll fluorescence technology has become one of the most powerful and widely used techniques used to understand the photosynthetic processes and thus, serve as a reliable indicator of stress in plants.^71^ Therefore, this study used this technique to analyze the effects of flooding stress on the activities of PSII and PSI in mulberry leaves. The results showed that although the *F*_v_/*F*_m_ of leaves began to decrease on the fifteenth day of flooding, the photosynthetic indices *PI*_ABS_ and *PI*_total_ of PSII activity and PSI activity ∆*I*/*I*_o_, respectively, began to decrease significantly on the fifth day of flooding, indicating that the PSII and PSI of mulberry leaves were damaged by flooding stress and their activities decreased. Ezin et al., ^48^ and Zeng et al., ^36^ found that the *F*_v_/*F*_m_ of tomato (*Solanum lycopersicum* L.) and alfalfa (*Medicago sativa* L.) under flooding stress was significantly lower than that of the control. A study by Caroline et al.,^37^ indicated that the treatment of *Allophylus edulis* with flooding stress led to a gradual decrease in the photosynthetic performance index on the third day of flooding, which demonstrated that PSII was damaged. The decrease in photosynthetic activity during flooding stress was also related to PSII damage and photosynthetic electron transport.^32^ Larre et al.^72^ found that the duration of flooding stress affects the damage of PSII structure and activity, as well as the development of plant photosynthetic regulation. To analyze the state of PSII in mulberry leaves under flooding stress in more detail, we standardized the original OJIP curve.^73^ The results showed that *V*_J_ and *V*_K_ in mulberry leaves increased significantly on the fifth day of flooding stress compared with the CK, and *V*_L_ increased on the tenth day of flooding stress. An increase in *V*_J_ and *V*_K_ is a sign of impaired electron transfer on the recipient side of PSII and impaired OEC on the donor side of PSII, respectively, while the increase in *V*_L_ reflects the dissociation of thylakoid membrane from PSII.^74^ Therefore, on the fifth day of flooding, electron transfer on the PSII acceptor side of mulberry leaves was blocked, and OEC on the donor side was damaged. Subsequently, on the tenth day of flooding, the thylakoid membrane of mulberry leaves was damaged. The OEC is primarily composed of *PsbO, PsbP* and *PsbQ*. In this experiment, five of the six coding genes located on the OEC were significantly downregulated. Simultaneously, other coding genes located at the PSII reaction center and those located at the PSI reaction center, including the genes for Cytb6/f, PC, Fd, FNR and ATP, were also significantly downregulated. PSI, PSII, Cytb6/f, PC, Fd, FNR and F-type ATPase are key components in the photosynthetic pathway. The genes related to leaf photosynthetic pathways were downregulated, including PSII, PSI, Cytb6/f, PC, FD, and the ATP synthesis machine among others, in the leaves of rape (*Brassica napus* L.) seedlings flooded for 3 days^68^ and soybean (*Glycine max* L. William 82 genotype) leaves flooded for 7 days.^52^ However, the upregulation of photosynthesis-related genes was observed in the early stress response of *Arabidopsis thaliana* under flooding stress for 3 h and *Populus tomentosa* under flooding stress for 5 h.^75^ Therefore, flooding stress promoted the expression of photosynthetically related genes during the initial stress response but inhibited them at the later stage.

Approximately 95% of the accumulation of plant biomass is owing to photosynthesis, and the inhibition of photosynthesis under stress is one of the important reasons that plant growth is hindered. Flooding stress was found to result in a decrease in photosynthesis, which manifested as a decrease in the CO_2_ concentration in leaves, which was caused by the limitation of stomata on gas exchange.^76^ The first response to flooding stress is stomatal closure,^77^ followed by a severe decrease in photosynthesis, respiration and transpiration rates.^78^ In this study, the *P*_n_, *G*_s_ and *T*_r_ of mulberry leaves decreased 10 days after flooding, and *C*_i_ also decreased 10 days after flooding. At this time, stomatal factors were the primary factors in the decrease of photosynthetic rate.^79^ Barickman et al. ^35^ also found that the gas exchange parameters of the leaves decreased, and photosynthetic activity was damaged in cucumber under flooding stress. In a study by Zafar et al., ^2^ flooding stress also reduced the gas exchange parameters (*G*_s_ and *C*_i_) and photosynthetic activity of muscadine grape (*Muscadinia rotundifolia* Michx.) leaves. In their study, the drop in photosynthetic rate was also owing to stomatal factors. Flooding has a serious impact on stomatal conductance and low CO_2_ assimilation, which is generally considered to be one of the most significant causes of reduced photosynthesis. However, this is not the only reason. On the fifteenth day of flooding, although the *C*_i_ was still lower than that of the CK, it increased significantly compared with the CK on tenth day. At this time, the decrease in photosynthetic rate was primarily caused by non-stomatal factors. In addition, the Calvin cycle in this study also confirmed that the downregulation of dark response genes also mediated a decrease in the photosynthetic capacity. The Calvin cycle is a part of the photosynthetic dark reaction, also known as the photosynthetic carbon cycle. RuBisCo is a key enzyme in the Calvin cycle, which participates in both photosynthesis and photorespiration and regulates the relationship between the two. RuBisCo is a key enzyme in the carbon assimilation of all photosynthetic organisms and catalyzes the fixation of CO_2_ to RuBP.^80^ PRK participates in the regeneration stage of the Calvin cycle and catalyzes the formation of RuBP from ribulose 5-phosphate.^81^ ALDO catalyzes the synthesis of FBP and RuBP in the Calvin cycle^[86]^. In this study, the levels of expression of the *Rubisco, PRK* and *ALDO* genes in mulberry leaves under flooding stress were significantly downregulated. Chen et al. ^53^ also found that flooding stress inhibited the expression of the *Rubisco* gene in soybean. PGK, GAPDH, TPI, FBPase, TK and R5PI are all key enzymes in the Calvin cycle pathway, which play an important role in the Calvin cycle. Under flooding stress, the levels of expression of their coding genes were the same as those of the *Rubisco* genes.

## 4. Conclusion

Flooding stress inhibited chlorophyll synthesis and decreased the content of Chl in mulberry leaves, and the sensitivity of Chl *a* to flooding stress was higher than that of Chl *b* owing to the changes in the genes *CHLG* and *CAO* that encode their products during chlorophyll synthesis. Simultaneously, the levels of expression of the *NYC, PPH* and *PAO* genes involved in chlorophyll degradation began to be upregulated on the fifteenth day of flooding, which accelerated the transformation of Chl *b* to Chl *a* and the degradation of Chl *a*. LHCII and LHCI identified seven and five antenna protein coding genes, respectively, that were significantly downregulated on the fifth day of flooding. The degree of downregulation was aggravated with the prolongation of flooding stress time, i.e., the degradation of chlorophyll and the downregulation of antenna protein coding genes inhibited the capture of light energy by mulberry leaves. The activity of PSII and PSI in mulberry leaves was decreased by flooding stress. On the fifth day of flooding, electron transfer on the PSII acceptor side of mulberry leaves was blocked, and OEC on the donor side was damaged. On the tenth day of flooding, the thylakoid membrane of mulberry leaves was damaged. Five of the six coding genes located on OEC were significantly downregulated. Simultaneously, other coding genes located in the PSII reaction center and coding genes located in the PSI reaction center, including cytb6/f, PC, FD, FNR and ATP, were also significantly downregulated. In addition, the gas exchange parameters of the leaves also decreased significantly after flooding stress. The stomatal factor was the main factor that resulted in the inhibition of photosynthesis during 10 d of flooding. However, on the fifteenth day of flooding, the joint restriction of stomatal and non-stomatal factors and the downregulation and expression of genes related to the Calvin cycle were the main limiting factors. In conclusion, the inhibition of mulberry plant growth caused by flooding stress was primarily related to the inhibition of chlorophyll synthesis, antenna proteins, photosynthetic electron transfer and the Calvin cycle. This study provides new insights for understanding the molecular mechanism of mulberry response and adaptation to flooding stress, and provides a theoretical basis for rational utilization and popularization of mulberry in flooded areas.
